# Primary cutaneous B-cell lymphoma leg type: good response with the RADHAP 21 protocol^⋆^

**DOI:** 10.1016/j.abd.2020.09.014

**Published:** 2022-01-15

**Authors:** Camila Gonçalves Pinheiro, Lafayette Cavalcanti Bezerra Dias Cruz, Alexandre Rolim da Paz, Luciana Cavalcante Trindade

**Affiliations:** a Dermatology Service, Faculdade de Medicina Nova Esperança, João Pessoa, PB, Brazil; b Oncology Service, Hospital Napoleão Laureano, João Pessoa, PB, Brazil; c Centro de Diagnóstico Anatomopatológico, João Pessoa, PB, Brazil

**Keywords:** Skin neoplasms, Lymphoma, Pharmacological treatment, Radiotherapy, Rituximab

## Abstract

Primary cutaneous lymphomas are defined as the ones that exclusively affect the skin for up to 6 months after the diagnosis. B-cell lymphomas represent 20-25% of primary cutaneous lymphomas and have, among its subtypes, the leg type, which represents 10 to 20% of cutaneous B-cell lymphomas, generally affecting elderly people and with an intermediate prognosis. This is the report of a rare case of a leg-type B-cell lymphoma with an exuberant clinical presentation affecting a young male patient.

## Introduction

Primary cutaneous lymphomas are neoplasms that appear on the skin without concomitant involvement of other organs for up to 6 months after the diagnosis. They show a wide clinical, immunophenotypic, histopathological, and prognostic diversity.^1^

Primary cutaneous B-cell lymphomas account for 20%–25% of all primary cutaneous lymphomas.^1^, ^2^ In the current World Health Organization and European Organization for Research and Treatment of Cancer (WHO-EORTC) classification, the primary cutaneous B-cell lymphoma, leg type has an intermediate prognosis, accounting for 10% to 20% of all primary cutaneous B-cell lymphomas.^3^ It usually affects older adults in the 7th decade of life.^4^ The diagnosis is attained through the association of clinical assessment, histopathological, immunohistochemical, and molecular biology analysis.^1^ The treatment is based on chemotherapy, which can be combined with radiotherapy.

The aim of this article is to report the case of a primary cutaneous B-cell lymphoma, leg type, affecting a young male patient due to its exuberant clinical presentation and the rarity of the diagnosis.

## Case report

**Figure 1:**
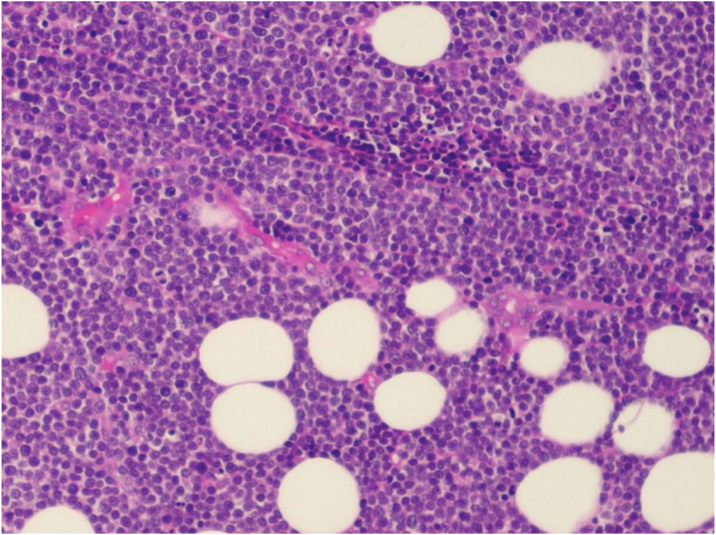
Large cell non-Hodgkin lymphoma infiltrating the inguinal lymph node, extending to the adipose tissue around the lymph node (Hematoxylin & eosin, ×200).

**Figure 2:**
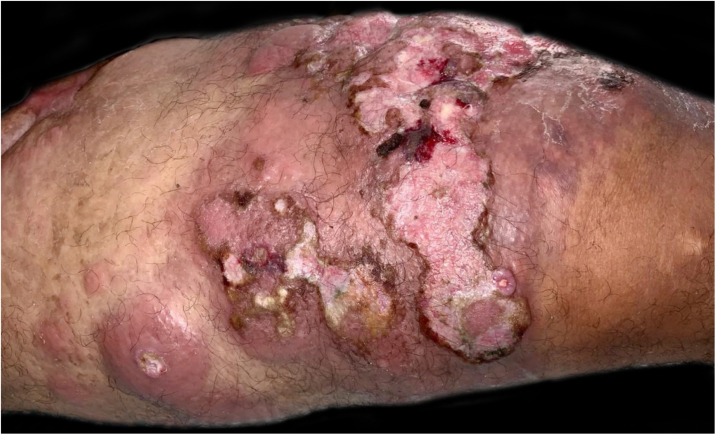
Infiltrated and vegetative tumors, grouped in a plaque with areas of ulceration, on the anterior and inner sides of the right thigh.

**Figure 3:**
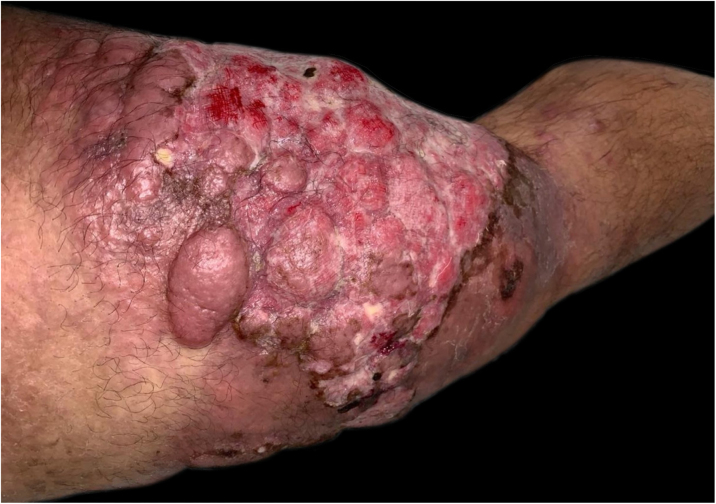
Relapsed infiltrated and vegetative tumors, grouped in a plaque with areas of ulceration, on the posterior and inner sides of the right thigh.

**Figure 4:**
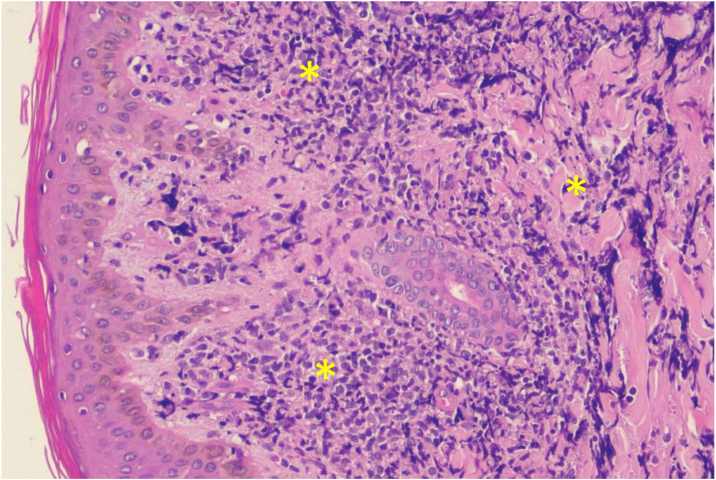
Histopathology of a skin fragment of the thigh. Neoplastic lymphocytes (*) diffusely infiltrating the dermis (Hematoxylin & eosin, ×200).

**Figure 5:**
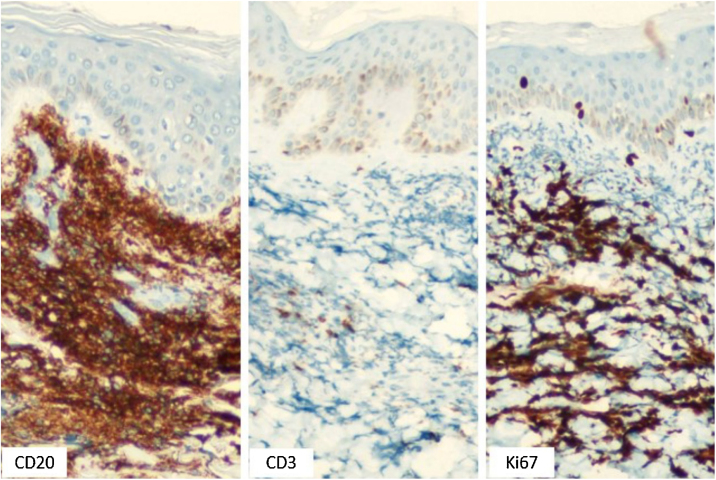
Skin fragment immunohistochemistry (×200): diffusely CD20-positive neoplastic cells with a high proliferative index (Ki67), in addition to rare residual non-neoplastic CD3+ “T” lymphocytes (Immunoperoxidase, ×200).

A 43-year-old male patient was seen in 2018 at a tertiary oncology service, where he was diagnosed with diffuse large B-cell non-Hodgkin's lymphoma, based on a right inguinal lymph node biopsy, positive for CD20 (L26), bcl-6 (LN22) and a Ki-67 (30-9) of 80% (Fig. 1). On that occasion, he received chemotherapy treatment with rituximab, doxorubicin, cyclophosphamide, vincristine, and prednisone (R-CHOP) and adjuvant radiotherapy, with disease remission documented by PETSCAN. After seven months, he reported the appearance of a nodular, asymptomatic, single lesion on the right thigh, which showed rapidly progressive growth. It developed into several tumors and vegetating painful lesions of varied sizes, affecting the entire diameter of the right thigh, which ulcerated and coalesced, forming an extensive plaque, with focal bleeding, fibrinoid tissue, and a fetid odor (Figure 2, Figure 3). He denied weight loss, night sweats, or fever, and the staging excluded the involvement of other organs. He reported being diabetic. Serology for HIV 1 and 2 was non-reactive. The histopathological examination of the lesion on the right thigh, performed in October 2019, showed findings consistent with diffuse large B-cell lymphoma, leg type. The immunohistochemistry was positive for CD20 (L26) with a Ki-67 (30-9) of 80%, and negative for CD3 (2GV6; Figure 4, Figure 5).

**Figure 6:**
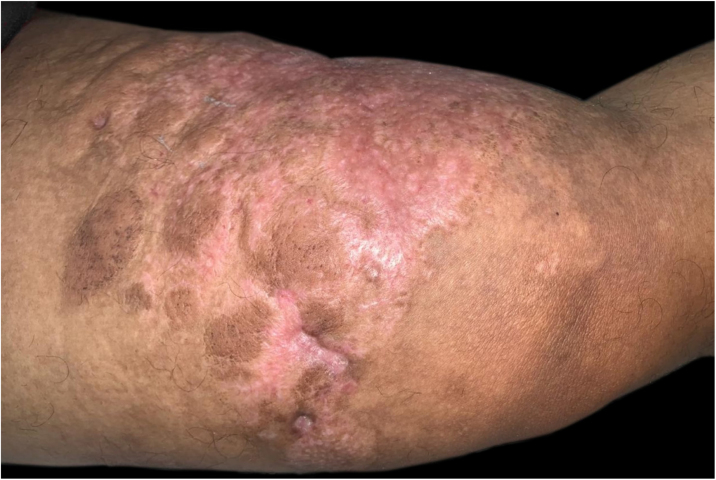
Regression of the tumors on the inner side of the right thigh after the 3^rd^ cycle of RADHAP chemotherapy.

He is currently undergoing chemotherapy treatment with the RADHAP 21 protocol, consisting of rituximab, cisplatin, and cytarabine. After the end of the three cycles, the patient showed excellent response, with lesion regression (Fig. 6). Due to the aggressiveness of the lymphoma and the rarity of the subtype, autologous bone marrow transplantation was also indicated.

## Discussion

The present report refers to a case of cutaneous B-cell lymphoma, leg type, with good therapeutic response. This histopathological type is unusual, being rare in young male individuals. Moreover, the present case stands out for its clinical exuberance.

Lymphomas are classified as primary cutaneous when they do not concomitantly affect other organs for up to 6 months after diagnosis. Although the patient had a previous diagnosis of lymphoma, the second diagnosis was considered to be a primary cutaneous tumor because at that time and until then, there was no involvement of other organs; thus, the two diagnosed lymphomas were considered independent of each other.

Primary cutaneous B-cell lymphoma (PCBCL), leg type, usually affects the lower limb and sporadically other sites such as the trunk, with a frequency of 7.5 to 13.3%. ^5^ It has an intermediate prognosis. It usually affects female individuals over 70 years old. Clinically, it presents as rapidly-growing erythematous-violaceous nodules or tumors, which may be single or multiple, clustered, ulcerated, located in one or both legs.^1^ The 5-year survival of patients varies from 36% to 55%, whereas in the other subtypes, it is 95%.^6^

In large series, extracutaneous dissemination was observed in 43% of patients, and it occurs mainly to lymph nodes, bone marrow, and central nervous system. Causal factors are not fully elucidated, but it is speculated that there may be a lymphoproliferative response to antigenic stimuli in the skin.^6^, ^7^ Some European studies have shown an association with *Borrelia burgdorferi* infection, but there are no similar data in the USA and Brazil.^2^

Histopathology reveals a dense infiltrate in the dermis and subcutaneous tissue, consisting of centroblasts and immunoblasts, which is separated from the epidermis by a collagen band, the Grenz zone. Epidermotropism is rare.^1^, ^4^ There may also be mitosis and a small number of reactive T lymphocytes, which are limited to perivascular areas.^8^

Neoplastic cells are positive for bcl-2, CD20, CD22 and CD79a. They are usually also positive for bcl-6, MUM-1 and FOXP1, while CD10 and CD138 are negative. The expression of bcl-2 confers a poor prognosis and may help in differentiating cutaneous B-cell lymphoma leg type from other types of CBCL.^4^, ^9^ Grange et al. (2007), in a study of 60 patients with CBCL leg-type, demonstrated that location in the lower limb and the presence of multiple lesions worsened the prognosis, with three-year survival rates of 43% in the leg subtype and 77% in the non-leg subtype.^10^

As for treatment, the first-line therapeutic options are chemotherapy with rituximab, doxorubicin, cyclophosphamide, vincristine, and prednisone (R-CHOP), with or without the addition of radiotherapy, but this approach is poorly documented. For patients who have a localized lesion or whose clinical condition does not allow aggressive treatments, monotherapy with rituximab or radiotherapy may be considered.^8^

The treatment indicated for the patient, in this case, was the RDHAP 21 protocol, with rituximab, cisplatin, and cytarabine, every 21 days, with regression of the lesions occurring after the third cycle, with a good therapeutic response to date.

## Financial support

None declared.

## Authors' contributions

Camila Gonçalves Pinheiro: Collection of data, drafting of the manuscript.

Lafayette Cavalcanti Bezerra Dias Cruz: Collection of data.

Alexandre Rolim da Paz: Collection of data.

Luciana Cavalcante Trindade: Critical review of the manuscript for important intellectual content, approval of the final version of the manuscript.

## Conflicts of interest

None declared.
